# Seropositivity for Chagas disease in blood donors from the state of Alagoas, Northeastern Brazil: an 11-year time series study

**DOI:** 10.1590/0037-8682-0339-2021

**Published:** 2021-12-17

**Authors:** Sílvia Letícia da Conceição Abreu, Márcio Bezerra-Santos, Flávia Silva Damasceno

**Affiliations:** 1 Universidade Estadual de Alagoas, Especialização em Doenças Parasitárias e Meio Ambiente, Santana do Ipanema, AL, Brasil.; 2 Universidade Federal de Sergipe, Hospital Universitário, Aracaju, SE, Brasil.; 3 Universidade Federal de Sergipe, Departamento de morfologia, Aracaju, SE, Brasil.; 4 Universidade de São Paulo, Instituto de Ciências Biomédicas, Departamento de Parasitologia, São Paulo, SP, Brasil.

**Keywords:** Chagas disease, Blood donors, Transfusional transmission disease

## Abstract

**INTRODUCTION:**

Chagas disease can be transmitted by blood transfusion. Herein, we assessed the seropositivity for Chagas disease in blood donors from Alagoas, during 2010-2020.

**METHODS:**

Data were requested from the Alagoas blood center. Time trend analysis was performed using a joinpoint regression model.

**RESULTS:**

Seropositivity rate during the study period was 0.35%, which decreased from 2014 to 2020, (annual percentage change, APC = -29.38; *p-*value <0.05), while the total number of tests performed remained stable (APC = 6.5).

**CONCLUSIONS:**

Despite the drop in the seropositivity for Chagas infection in donors, it is imperative to maintain the screening of donors for the epidemiological control.

Chagas disease (CD), also known as American trypanosomiasis, is caused by the protozoan parasite *Trypanosoma cruzi* (*T. cruzi*). The World Health Organization (WHO) estimates that 7 million people are affected by CD causing nearly 12,000 deaths annually. The disease is endemic in Latin American countries and the southwestern region of the United States^1^. Although CD cases mainly occur on the American continent, the geographical distribution of the disease is worldwide due to the migration of people from endemic to non-endemic areas. As a result, it is estimated that 70 million people worldwide are at risk of infection^2^ ^-^ ^4^.

Clinically, CD demonstrates both acute and chronic phases. The acute phase, occurring shortly after infection and commonly being asymptomatic, is characterized by the presence of parasites in the bloodstream with an absence of detectable specific antibodies. In contrast, the chronic phase is characterized by low concentration of parasites in the bloodstream and the presence of specific anti-*T. cruzi* IgG antibodies. During the chronic phase, most patients are asymptomatic throughout life; however, some patients experience symptoms, such as megaviceras and heart failure^2^ ^,^ ^4^. Importantly, most cases of CD are diagnosed in the chronic phase when the patients present symptoms, while the absence of or unspecific symptoms results in difficulty of diagnosis in the acute phase. Nonetheless, in Brazil, only acute cases of CD are reported to public health services.

The main form of transmission of *T. cruzi* in endemic areas is via insect vectors, specifically bedbugs of the subfamily Triatominae. Additionally, CD can be transmitted through ingestion of contaminated food, congenitally through pregnancy or during childbirth, via organ transplantation, during blood transfusion from an infected donor, or laboratory accidents^2^ ^,^ ^4^ ^,^ ^5^. 

Transfusion transmission of *T. cruzi* was first confirmed in the 1950s^6^ and was considered a relevant public health problem, leading to the implementation of a serological test for CD in blood banks in Brazil and other countries. The implementation of serological testing for CD gradually increased in the last decades, with the exception of some countries. Despite conducting serological tests for CD on donated blood, the risk of transfusion transmission is still present in Brazil. Recently, Santos et al. described that 0.31% of the acute CD cases notified in Brazil, in the two last decades, had transfusion as the probable route of infection^5^. Unfortunately, scientific articles relating to CD positivity rates of blood donated in Brazil are scarce in the articles database. In the Northeast Region, we can find these data only from the states of Ceará, Pernambuco, and Piauí, all of which demonstrate the occurrence of CD seropositive individuals among blood donors^7^ ^-^ ^9^. However, no information is available regarding CD seropositivity rates among blood donors in Alagoas. More importantly, there have been no cases of CD notified by the disease notification system of the Brazilian Ministry of Health^10^ in the last decade in Alagoas. Meanwhile, according to the Brazilian mortality information system^11^, the annual average of deaths from CD in the same period was approximately 100 per year. This lack of information makes it difficult to plan health policies to prevent and control CD in Alagoas. 

Considering the lack of information on CD in the State of Alagoas, the objective of the current study was to assess the rate of seropositivity for CD in blood donors in this endemic region of Northeast Brazil between 2010 and 2020. 

We carried out a time series study on data related to the total number of tests performed in the period between 2010 to 2020 in order to assess CD in blood samples and the number of seropositive tests to IgG anti-*T. cruzi* antibody. Data were requested from the Alagoas blood center, HEMOAL. This institution attends blood donors from urban and rural areas of the entire state of Alagoas, Northeastern Region of Brazil. Traditionally, HEMOAL utilized enzyme-linked immunosorbent assay (ELISA) as a diagnostic method to identify IgG anti-*T. cruzi* in blood samples. However, since 2013, the institution has relied on the chemiluminescence technique. Absolute and relative frequencies were calculated using Microsoft Excel (2017) software. 

Additionally, we assessed time trends contained within the data using the joinpoint regression model (segmented linear regression). The annual percentage change (APC) and its respective 95% confidence interval (95% CI) were calculated for each segmented period. Thereby, significant positive values of APC (*p*-value <0.05) indicate increasing trends over time, while significant negative APC values indicate a decreasing trend. Similarly, APC values that were not significantly different between time periods indicate a stationary trend^12^. Moreover, when a time trend had inflection points and more than one APC, we calculated the average annual percentage change (AAPC) for the entire period. Time trends were considered statistically significant when the APC or AAPC had a *p*-value <0.05 and was accompanied by a 95% CI that did not include a zero value. We used the Joinpoint Regression Program, version 4.5.0 (National Cancer Institute, Maryland, USA) for the analysis. 

A total of 322,060 blood tests for CD were performed by HEMOAL between 2010 and 2020, while 1,142 (0.35%) of these tests were positive for IgG anti-*T.cruzi* antibodies. Analyzing the total number of CD tests performed, a stable trend was observed between 2010 and 2020 (APC = 1.85) (Figure 1A and Table 1). Likewise, a stable trend over time was identified for positive tests for IgG anti-*T. cruzi* antibodies between 2010 and 2014 (APC = 6.5). However, a decreasing trend was observed from 2014 to 2020 (APC = -29.38*, *p*-value <0.05) (Figure 1B and Table 1). Similarly, a decreasing trend was seen when considering positive tests for IgG anti-*T. cruzi* antibodies over the entire time period (2010 = 148; 2020 = 23), (AAPC = -16.8; *p*-value <0.05). 

**FIGURE 1:**
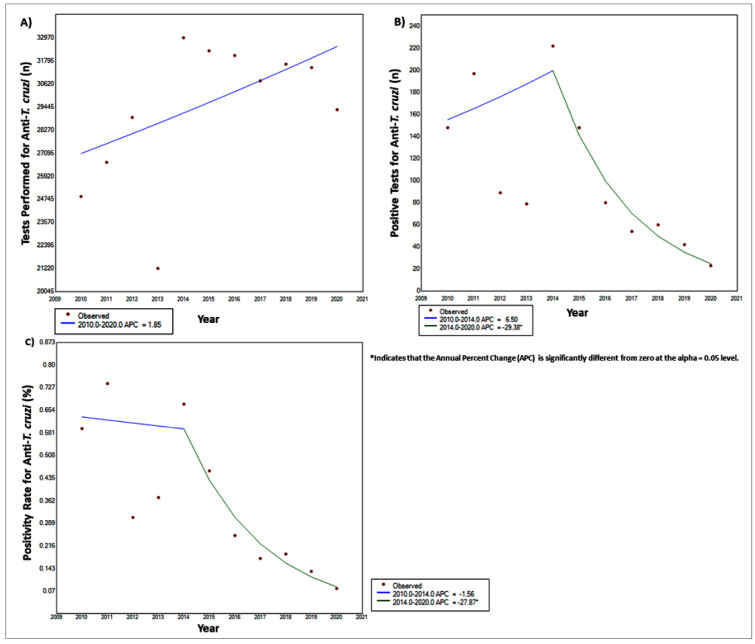
**Joinpoint temporal trend evaluation of seropositive tests for Chagas disease in the state of Alagoas, Northeastern Brazil, between 2010 and 2020.** Temporal trend analysis of: **(A)** Total number of anti-*T. cruzi* tests performed by Alagoas blood center - HEMOAL. **(B)** Positive tests for IgG anti-*T. cruzi*. **(C)** Positivity rate for IgG anti-*T. cruzi*. APC: Annual Percent Change. *p-value <0.05.

**TABLE 1:** Temporal trends in the positivity rate for anti-*T. cruzi* and number of tests performed by HEMOAL, state of Alagoas, Northeast Brazil, between 2010 and 2020.

Variables			Segmented Period				Total Period		
	2010	2020	Period	APC^a^ (*p <0.05)	95% CI	Trend	AAPC^b^ (*p <0.05)	95% CI	Trend
**Tests Performed for Anti-*T. cruzi* (n)**	24,886	29,300	2007-2016	1.85	-0.5, 4.3	Stable	--	--	--
**Positive Tests for Anti-*T. cruzi* (n)**	148	23	2010-2014	6.5	-23, 47.2	Stable	-16.8*	-30.4, -0.5	Decreasing
			2014-2020	-29.38*	-47.8, -4.5	Decreasing			
**Positivity Rate for Anti-*T. cruzi* (%)**	0.59	0.07	2010-2014	-1.56	-24.1, 27.6	Stable	-18.3*	-31.5, -2.5	Decreasing
			2014-2020	-27.87*	-47.9, -0.2	Decreasing			

^a^Annual Percent Change; ^b^Annual Average Percent Change; n = number of samples; *Indicates that the APC or AAPC is significantly different from zero at the alpha = 0.05 level.

In addition, we calculated the percentage of positive tests for CD related to the total number of tests performed in the analyzed period. As expected, the joinpoint analysis showed the same temporal profile regarding positivity rates for IgG anti-*T. cruzi*, corroborating the stable trend identified in the period from 2010 to 2014 (APC = -1.56) and the decreasing trend over time observed between 2014 and 2020 (APC = -27.87*; *p*-value <0.05). Similarly, there is a decreasing trend when considering the entire period of time analyzed (2010 = 0.59%; 2020 = 0.07%; AAPC = -18.3; *p*-value <0.05) (Figure 1C and Table 1). 

To our knowledge, this is the first study that addresses CD in blood donors from the state of Alagoas, an endemic area in Northeastern Brazil. Herein, we demonstrate that 0.35% of the blood donors in Alagoas were positive for IgG anti-*T. cruzi* antibody between 2010 and 2020. The WHO considers CD as a neglected tropical disease that mainly affects low-income populations worldwide, with the largest number of cases in Latin America^3^. Considering our findings and that Alagoas has the lowest Brazilian Human Development Index (HDI = 0.683)^13^, we speculate that CD transmission persists in this state, mostly in municipalities with high social vulnerability.

Remarkably, vector transmission is the most important route of CD transmission in endemic areas. However, other transmission routes remain important in both endemic and non-endemic areas, such as transfusional transmission. The transfusional transmission of CD was first confirmed in the 1950s^6^; this method of transmission occurs when a blood bank provides blood for transfusion supplied by a donor infected with *T. cruzi*. Currently, Brazilian blood centers control blood bags to avoid CD transmission. Nevertheless, more than half-century after the implementation of anti-*T. cruzi* tests in blood banks, the risk of CD transmitted by blood transfusion are still reported in Brazil. In the last two decades, 0.31% of the notified acute CD cases in Brazil, had transfusion as the probable route of infection^5^. 

In this study, we demonstrate that the positivity rate for CD among blood donors in Alagoas was 0.35%. This value is similar to the data reported from the State of Ceará (0.33% in 2015)^9^; while being less than the positivity rate reported in the State of Piauí from 2004 to 2013 (1%)^7^; and greater than in the Pernambuco State from 2002 to 2007 (0.17%)^8^. The WHO estimate of the seropositivity rate in blood donors from Brazil in 2010 was 0.18%^14^, while our results show the seropositive rate in Alagoas is two-fold more than that. 

Despite the positive tests for CD observed among blood donors between 2010 and 2020, surprisingly, there were no cases of CD notified in Alagoas by the DATASUS during this same period; meanwhile, approximately 100 deaths were notified to be caused by CD annually in this state^10^ ^,^ ^11^. We highlight that, in general, blood donors consist of people who feel healthy, without fever or discomfort, which suggest that the seropositive blood donors were in the later acute phase or asymptomatic chronic phase of CD when donating. Moreover, once a blood donor is diagnosed with CD or any other transmissible illness, they are prevented from donating blood again^15^. Therefore, even with the definitive control of the transmission of the infection in the state of Alagoas, since the asymptomatic carrier of the infection has harbored the parasite for several decades, and blood donation in the country can be done up to 70 years of age, the occurrence of infected donors could continue for years; thus, requiring the maintenance of control measures. 

Our data demonstrate that, despite the decreasing trend of seropositive tests, it remains necessary to carry out these tests on all the donated blood before it is used for transfusion in order to prevent the transmission of CD via blood or blood products. 

Our study presented some limitations; it was not possible to access data related to sex, age, socioeconomic characteristics, the municipality of blood donor residence, and donor characteristics related to number of donations. 

Regardless of the decreasing trend in the positivity rate for IgG anti-*T. cruzi* antibodies in blood donors, our data suggest that transfusional transmission of CD continues to be a risk, which justifies the need to maintain the testing of samples of the donated blood in the state of Alagoas. 

## ETHICAL STATEMENT

We only used secondary data; thus, the project does not need to be submitted to the ethics and research committee. The databank used in the study does not allow identification of patients.
